# A Redox-Sensitive Thiol in Wis1 Modulates the Fission Yeast Mitogen-Activated Protein Kinase Response to H_2_O_2_ and Is the Target of a Small Molecule

**DOI:** 10.1128/MCB.00346-19

**Published:** 2020-03-16

**Authors:** Johanna J. Sjölander, Agata Tarczykowska, Cecilia Picazo, Itziar Cossio, Itedale Namro Redwan, Chunxia Gao, Carlos Solano, Michel B. Toledano, Morten Grøtli, Mikael Molin, Per Sunnerhagen

**Affiliations:** a University of Gothenburg, Department of Chemistry and Molecular Biology, Gothenburg, Sweden; b Chalmers University of Technology, Department of Biology and Biological Engineering, Gothenburg, Sweden; c Oxidative Stress and Cancer Laboratory, Integrative Biology and Molecular Genetics Unit, CEA Saclay, Gif-sur-Yvette, France

**Keywords:** Sty1, cysteine oxidation, stress signaling, *Schizosaccharomyces pombe*

## Abstract

Oxidation of a highly conserved cysteine (Cys) residue located in the kinase activation loop of mitogen-activated protein kinase kinases (MAPKK) inactivates mammalian MKK6. This residue is conserved in the fission yeast Schizosaccharomyces pombe MAPKK Wis1, which belongs to the H_2_O_2_-responsive MAPK Sty1 pathway. Here, we show that H_2_O_2_ reversibly inactivates Wis1 through this residue (C458) *in vitro*. We found that C458 is oxidized *in vivo* and that serine replacement of this residue significantly enhances Wis1 activation upon addition of H_2_O_2_. The allosteric MAPKK inhibitor INR119, which binds in a pocket next to the activation loop and C458, prevented the inhibition of Wis1 by H_2_O_2_ *in vitro* and significantly increased Wis1 activation by low levels of H_2_O_2_ *in vivo*. We propose that oxidation of C458 inhibits Wis1 and that INR119 cancels out this inhibitory effect by binding close to this residue. Kinase inhibition through the oxidation of a conserved Cys residue in MKK6 (C196) is thus conserved in the S. pombe MAPKK Wis1.

## Abstract (precis)

Oxidation of a highly conserved cysteine (Cys) residue located in the kinase activation loop of mitogen-activated protein kinase kinases (MAPKK) inactivates mammalian MKK6. This residue is conserved in the fission yeast Schizosaccharomyces pombe MAPKK Wis1, which belongs to the H_2_O_2_-responsive MAPK Sty1 pathway. Here, we show that H_2_O_2_ reversibly inactivates Wis1 through this residue (C458) *in vitro*.

## INTRODUCTION

Mitogen-activated protein kinases (MAPKs) are evolutionarily conserved kinases that operate in a three-tier kinase cascade module that comprises the MAPK, which is activated by phosphorylation on juxtaposed Tyr and Ser residues by a dual-specificity MAPK kinase (MAPKK). A MAPKK requires activation through phosphorylation by a MAPKK kinase (MAPKKK). MAPKs regulate cellular responses by phosphorylating transcription factors and other kinases (1).

The Schizosaccharomyces pombe Sty1 kinase is the homolog of the mammalian stress-activated MAPK p38. Like p38, Sty1 responds to external stress stimuli, including heat, osmotic and acidic stresses, metals, UV-induced DNA damage, and hydrogen peroxide (H_2_O_2_) (2). In the Sty1 pathway, the H_2_O_2_ signal is integrated at the level of a membrane-bound two-component phosphorelay system, including the histidine kinases Mak2 and Mak3 (Mak2/3) (3). Mak2/3 relay the H_2_O_2_ signal to the MAPKKKs (4) Win1 (5) and Wis4/Wik1/Wak1 (6) by phosphate transfer on an aspartic residue of the Mcs4 response regulator via the phosphorelay protein Mpr1. MAPKKKs, in turn, activate the MAPKK Wis1 by phosphorylation on S469 and T473. Wis1, the homolog of the mammalian MAPKK MEK1, activates the MAPK Sty1 (7) by dual phosphorylation on T171 and Y173 (8). Sty1 is the only known target of Wis1. When active, Sty1 phosphorylates the Aft1 transcription factor, which regulates a transcriptional response to stress. Similar to the Sty1 pathway, the human p38 MAPK pathway is activated by H_2_O_2_ stress (9).

**FIG 1:**
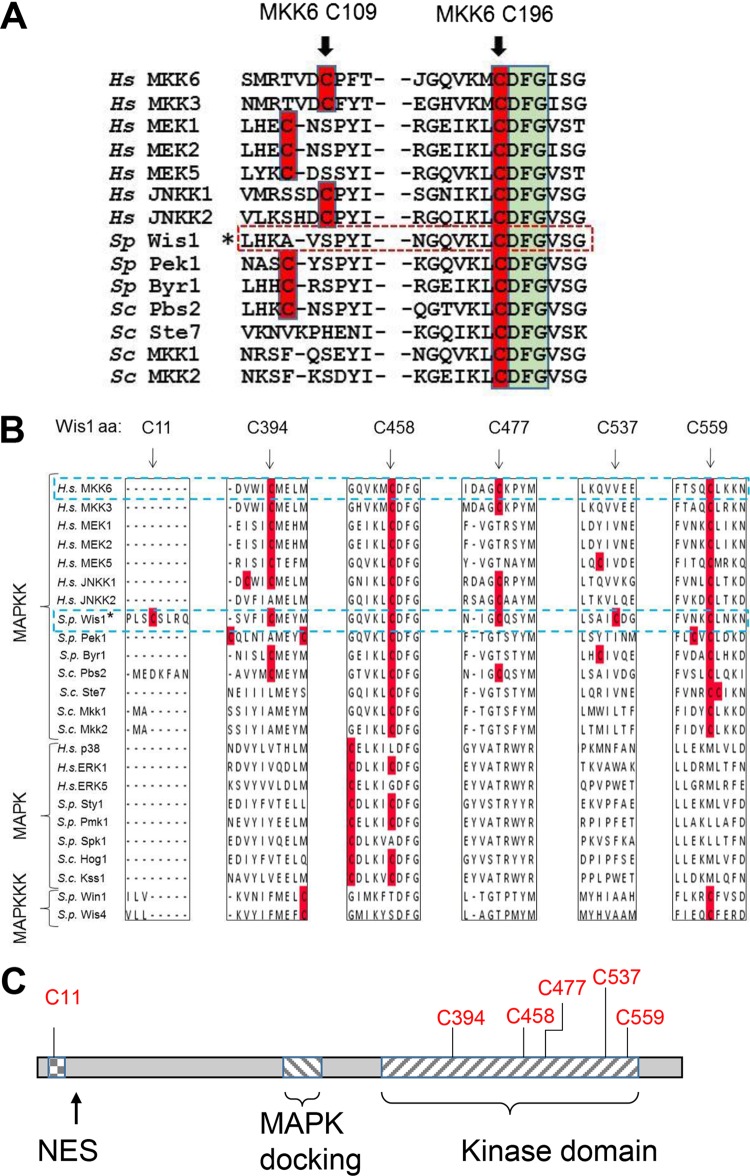
Wis1 contains a conserved cysteine next to the DFG motif but lacks a cysteine homologous to MKK6C109. (A) Multiple alignment of the human, fission yeast, and budding yeast MAPKKs showing conservation of the cysteines involved in inhibition through disulfide formation in human MKK6. Cysteines are highlighted in red, the Wis1 sequence is marked with an asterisk, and the DFG motif is indicated by a green box. MKK6 Cys196/Wis1 Cys458 directly precedes the highly conserved DFG motif and is conserved in all MAPKKs, whereas the position of MKK6 Cys106 is less conserved. Wis1 has no cysteine corresponding to MKK6 Cys106. (B) Multiple alignment showing the degree of conservation of all cysteines in Wis1. Human, fission yeast, and budding yeast MAPKKs, MAPKs, and MAPKKKs are shown aligned with the homologous sequences flanking the positions of the six Wis1 cysteines. Conservation is restricted to MAPKKs, except for C458 which is also present in some MAPKs. aa, amino acid. (C) Schematic overview of the location of cysteines in Wis1 in relation to functional information within the Wis1 amino acid sequence. Five of the total six cysteines are found within the kinase domain, and one is found within the nuclear export signal (NES). The locations of functional domains in this figure are based on the work of Nguyen et al. (44). Hs, Homo sapiens; Sc, Saccharomyces cerevisiae; Sp, Schizosaccharomyces pombe.

Even though the p38 pathway is activated by H_2_O_2_, one of the p38 MAPKKs, MKK6, becomes inactivated at cell exposure to low doses of H_2_O_2_ through the formation of a disulfide bond between a Cys residue, evolutionarily conserved among MAPKKs at position −1 of the DFG motif in the kinase activation loop (C196), and another conserved residue (C109) (Fig. 1A), which inhibits ATP binding (10). The aspartate residue of the DFG motif coordinates Mg^2+^, thereby contributing to the phosphotransfer reaction from ATP (11). The MKK6 C196 residue is conserved in all MAPKKs, including Wis1 and the Saccharomyces cerevisiae Wis1 homolog Pbs2, but not in other S/T kinase families, and it may have a conserved redox function.

Here, we have examined the possible regulatory role of the S. pombe Wis1 C458 residue, which corresponds to MKK6 C196 and is the third cysteine of six from the N terminus (Fig. 1B and C). We found that similar to human MKK6, Wis1 is inactivated by H_2_O_2_ through reversible oxidation, both *in vitro* and *in vivo*, and in a manner dependent on the presence of the C458 residue. We used the non-ATP competitive allosteric MEK1 inhibitor INR119 (12), which binds MEK1 in a pocket also conserved in Wis1, close to C458, as a tool to investigate the regulatory role of this cysteine. We found that INR119 enhances Wis1 kinase activity by canceling out the kinase inhibition exerted by C458 oxidation. Last, we observed that Wis1 C458 is crucial for cellular tolerance to H_2_O_2_ but not to KCl, consistent with the need of appropriate, dose-dependent Sty1 pathway activation upon stress.

(A substantial portion of this research was conducted by Johanna J. Sjölander in partial fulfillment of the requirements for a Ph.D. from the University of Gothenburg, Gothenburg, Sweden, 2019.)

## RESULTS

### H_2_O_2_ inhibits Wis1 kinase activity by oxidation of C458.

In mammals, H_2_O_2_ reversibly inhibits the p38 MAPK MKK6 by causing the formation of a disulfide bond between C109 and C196 (10). C196 is located in the kinase-activating loop, directly upstream (−1 position) of the highly conserved DFG kinase motif, which binds Mg^2+^ and thereby contributes to catalysis. C196 is conserved in mammalian MAPKKs, in the S. pombe and S. cerevisiae MAPKKs Wis1 and Pbs2, respectively, and in several MAPKs (Fig. 1A) (11). The S. pombe MAPKK Wis1 carries the residue corresponding to C196 in MKK6 at position 458 but lacks the MKK6 C109 residue. We examined whether Wis1 C458 is a site of redox regulation.

**FIG 2:**
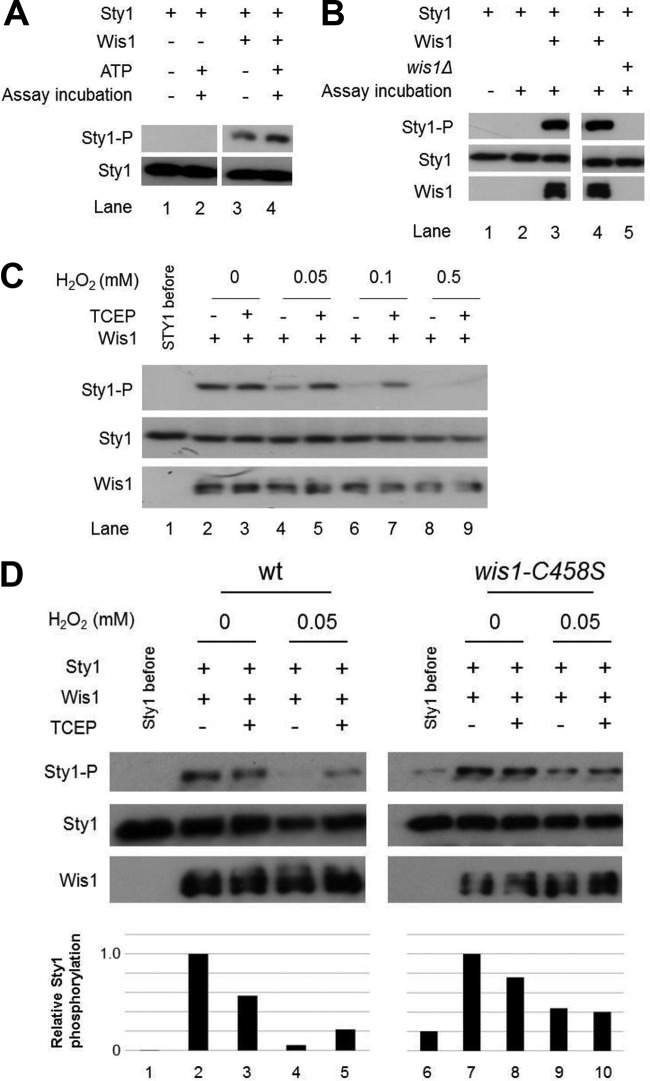
Wis1 is inhibited by oxidation of Cys458 *in vitro*. (A) *In vitro* Wis1 kinase activity is constitutive upon coincubation of Wis1 and Sty1 purified in the absence of EDTA, suggesting that ATP copurifies with Wis1. Sty1 is not phosphorylated before (lane 1) or after (lane 2) assay incubation without Wis1 added. However, even without addition of ATP to the reaction buffer (lane 3), Wis1 phosphorylates Sty1 to the same degree as when ATP is included (lane 4). (B) Wis1 purified in the presence of EDTA phosphorylates Sty1 in an Mg^2+^- and ATP-dependent manner *in vitro*. Precipitates of Ni^2+^ beads from strain JJS7 expressing HA-Wis1-His_6_ or *wis1Δ* (JJS1) were tested for activity against the substrate Sty1. No phosphorylation was seen when everything except HA-Wis1-His_6_ from strain JJS7 (lane 2) was added or when the precipitates from strain JJS7 were exchanged with a precipitate from strain JJS1 (*wis1Δ*, lane 5). Note that no H_2_O_2_ was included in this assay. (C) Inactivation of Wis1 by low levels of H_2_O_2_ *in vitro* is caused by reversible thiol oxidation. Semipurified Wis1 (strain JJS7) was first incubated for 5 min in sample pairs with H_2_O_2_, giving the indicated final concentration, and subsequently for one tube in each pair for 5 min with 1 mM TCEP. Thereafter the Wis1 substrate Sty1 (expressed from pREP41MHNSTY1 in strain KS1598) was added, and the kinase reaction was started by addition of MgCl_2_-ATP. The kinase reaction was stopped after 20 min. (D) Wis1 is inactivated by low levels of H_2_O_2_ *in vitro* in a manner reversible by the reductant TCEP. In contrast, a Wis1^C458S^ mutant enzyme is less inactivated, and activity lost could not be rescued by reductant. Wis1 was treated for 5 min with H_2_O_2_ before the kinase reaction was starting by the addition of MgCl_2_-ATP. This inactivation was in turn reversed upon a 5-min incubation with the reductant TCEP at 1 mM before the kinase assay. Wild-type Wis1 was expressed from JJS7, and Wis1^C458^ was expressed from JJS9. The bottom panel shows quantification of the relative levels of Sty1 phosphorylation in the different lanes.

We first inquired whether Wis1 kinase activity is modulated by H_2_O_2_ *in vitro*, using Wis1 and Sty1 purified from two different S. pombe strains (see Materials and Methods). When purified in the absence of EDTA, Wis1 phosphorylated Sty1, even without ATP addition (Fig. 2A), suggesting that Wis1 is ATP bound. When purified in the presence of EDTA and under nonreducing conditions, Wis1 phosphorylated Sty1 in an ATP- and Mg^2+^-dependent manner (Fig. 2B). Notably, when purified under these conditions, Wis1 no longer phosphorylated Sty1 when incubated with H_2_O_2_ for 5 min. The effect of H_2_O_2_ was dose dependent, visible at 50 μM and increased at 100 and 500 μM, and reversed by the thiol reductant tris(2-carboxyethyl)phosphine (TCEP) in samples exposed to H_2_O_2_ at 50 and 100 μM but not at 500 μM (Fig. 2C).

We next tested whether C458 is required for the inhibition of Wis1 by H_2_O_2_ using a Wis1 mutant with a serine substitution for C458 (Wis1^C458S^) (Fig. 2D, right panel). Wis1^C458S^ retained full kinase activity *in vitro*. H_2_O_2_ decreased this activity by about half but did not completely inhibit it as it did with the wild-type (wt) protein, and this partial inhibition was not reversed by TCEP.

We conclude that H_2_O_2_ reversibly inhibits Wis1 kinase activity in a manner dependent on C458, which suggests that this residue becomes oxidized, possibly to a sulfenic (-SOH) or disulfide bond form. Moreover, the partial and irreversible inhibition of Wis1^C458S^ suggests that one or more additional targets of H_2_O_2_ in Wis1 impinge on its activity.

### Wis1 C458 is oxidized *in vivo*.

**FIG 3:**
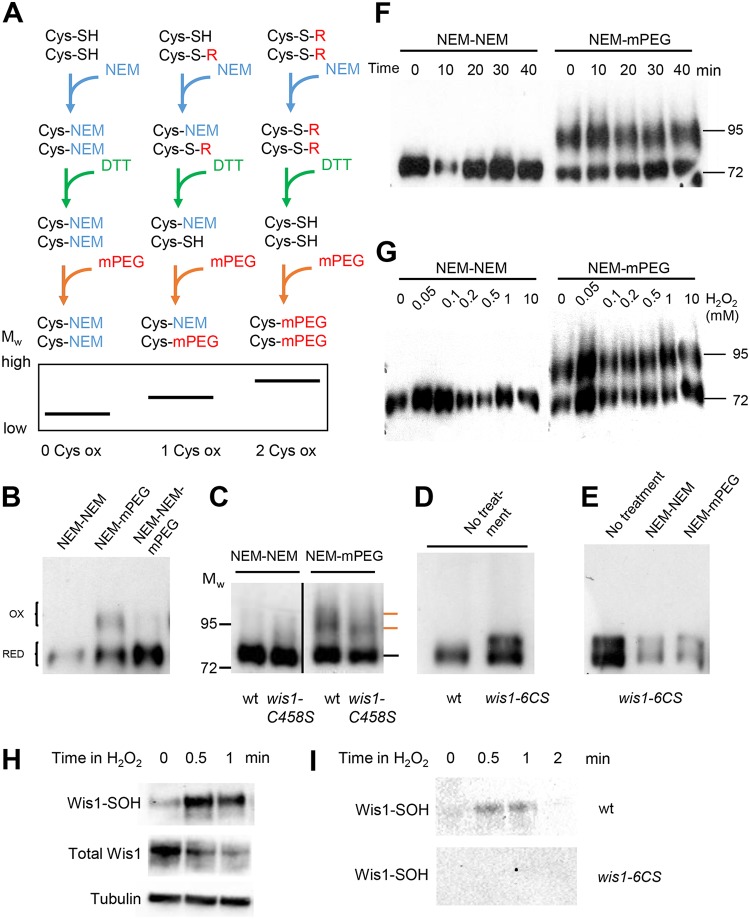
Wis1 cysteines including C458 are oxidized *in vivo*, and Wis1 is rapidly sulfenylated upon H_2_O_2_ addition. (A) Schematic overview of the mPEG assay. NEM alkylates all presently reduced thiols. DTT reduces the remaining (reversibly oxidized [ox]) thiols, making them accessible for alkylation by mPEG. This adds approximately 5 kDa per oxidized thiol. Note that irreversibly oxidized thiols not reduced by DTT will not be mPEG labeled. MW, molecular weight. (B to G) The mPEG assay shows that Wis1 C458 as well as other cysteines in Wis1 are oxidized *in vivo* even without exogenous H_2_O_2_. Cells analyzed were from strain JJS15 expressing HA-tagged wt Wis1 from the endogenous promoter (B to D, F, and G), from strain JJS16 expressing cysteine-less Wis1^6CS^ from the endogenous promoter (C and D), or from strain JJS7 and JJS9 expression wt Wis1 and *wis1^C458S^*, respectively, from the *nmt1* promoter (E). (B) Slower-migrating mPEG-modified Wis1 bands (marked with orange lines) appear in extracts of cells even without H_2_O_2_ treatment. Formation of these slower-migrating forms is blocked by an extra round of NEM before mPEG treatment. RED, reduced. (C) mPEG assay of wt and *wis1^C458S^* cell extracts without H_2_O_2_ added. The upper of the two bands representing oxidized Wis1 (orange markers) is absent in the strain expressing Wis1^C458S^ mutant protein. (D and E) The constitutively mPEG-dependent altered migration of Wis1 is abolished in a cysteine-less mutant Wis1 protein (Wis1^6CS^). A strain expressing cysteine-less Wis1^6CS^ was run on the blot together with wt Wis1 (B) or after the mPEG assay (C). Samples were divided in two equal aliquots and subjected to either NEM-DTT-NEM treatment or NEM-DTT-mPEG. (F) Time course of Wis1 cysteine oxidation in wt cells as assayed by the mPEG assay following the addition of 0.5 mM H_2_O_2_ for the indicated duration. (G) Wis1 cysteine oxidation as assayed by the mPEG assay at 20 min after the addition of H_2_O_2_ to different concentrations. (H and I) The use of a sulfenylation-specific probe shows rapid cysteine-dependent Wis1 sulfenylation following H_2_O_2_ addition. Cells expressing HA-tagged wt Wis1 (JJS15) (H and I) or Wis1^6CS^ (JJS16) (I) were labeled with the sulfenyl-binding reagent DYn-2 prior to exposure to 0.5 mM H_2_O_2_, and sample withdrawal was performed at 30 s and 1 and 2 min thereafter. Sulfenylated protein was immunoprecipitated as described in Materials and Methods. Following separation by SDS-PAGE, sulfenylated Wis1 was detected with anti-HA antibodies.

We inquired whether Wis1 becomes oxidized *in vivo* upon cell exposure to H_2_O_2_ by differentially labeling reduced and oxidized Cys residues with *N*-ethylmaleimide (NEM) and methoxy polyethylene glycol (mPEG) 5000, respectively (Fig. 3A). mPEG adds 5 kDa per oxidized Cys residue, thereby decreasing protein electrophoretic mobility in proportion to the number of residues modified. We first used lysates from cells not exposed to H_2_O_2_. Migration of hemagglutinin (HA)-tagged Wis1 was partially shifted by mPEG to a doublet band of slower migration (Fig. 3B), thus indicating that in exponentially growing cells, one or more Wis1 Cys residues are already partially oxidized into a form sensitive to reduction by dithiothreitol (DTT). The Wis1^C458S^ mutant protein was also shifted, but its band shift lacked the upper band of the doublet, therefore indicating that this residue contributes to the oxidation seen in the wt protein in the absence of exogenous H_2_O_2_ addition (Fig. 3C). As a control, a *wis1* mutant lacking all six Cys residues (*wis1^6CS^* strain, with all Cys residues replaced with Ser) did not display any electrophoretic mobility shift after mPEG derivatization, therefore confirming that the mPEG gel shifts seen with wt and mutant Wis1 proteins are caused by mPEG derivatization at Cys residues (Fig. 3D and E). For unclear reasons, the Wis1^6CS^ mutant protein migrated as a double band, in a manner independent of mPEG derivatization. We next monitored the effect of cell exposure to H_2_O_2_. Surprisingly, H_2_O_2_ did not change the pattern of migration of mPEG-derivatized Wis1, as monitored in a time course analysis at time points relevant to maximal Wis1 signaling and upon the addition of 500 μM H_2_O_2_, or in a range from very low (50 μM) to very high (10 mM) doses (Fig. 3F and G). We conclude that C458 is oxidized *in vivo* even without the addition of H_2_O_2_.

The lack of a net change in Wis1 cysteine oxidation upon H_2_O_2_ addition, as seen by the mPEG-cysteine derivatization assay, suggested that exogenous H_2_O_2_ does not further oxidize Wis1. The initial step in cysteine oxidation by H_2_O_2_ is the conversion of the reduced cysteine (-SH) into a sulfenic acid, Cys-SOH. Cysteine sulfenic acids are very transient due to their instability (13) but were recently shown to form readily upon H_2_O_2_ treatment in several species (14). DYn-2 [4-(pent-4-yn-1-yl)cyclohexane-1,3-dione] is a sulfenic acid-specific dimedone derivative, and its use *in vivo* increases the detection of this unstable species (15). Using this strategy, we found that Wis1 labeled with DYn-2 already prior to cell exposure to H_2_O_2_ (Fig. 3H and I). Furthermore, the sulfenic acid signal strongly increased 30 s following cell exposure to 0.5 mM H_2_O_2_ and then decreased back to the levels seen prior to H_2_O_2_ addition after 2 min (Fig. 3H and I), thus indicating the presence of at least one sulfenylated cysteine residue. The two approaches provide seemingly contradictory results, but it must be pointed out that they do not monitor cysteine oxidation in the same time frame and that the DYn-2-based method is far from quantitative. Accordingly, these data can be interpreted in the way that at least two cysteines are oxidized in Wis1 prior to H_2_O_2_ addition, of which at least one is in the sulfenylated form, and one or more cysteines in Wis1 become transiently sulfenylated upon H_2_O_2_ addition.

**FIG 4:**
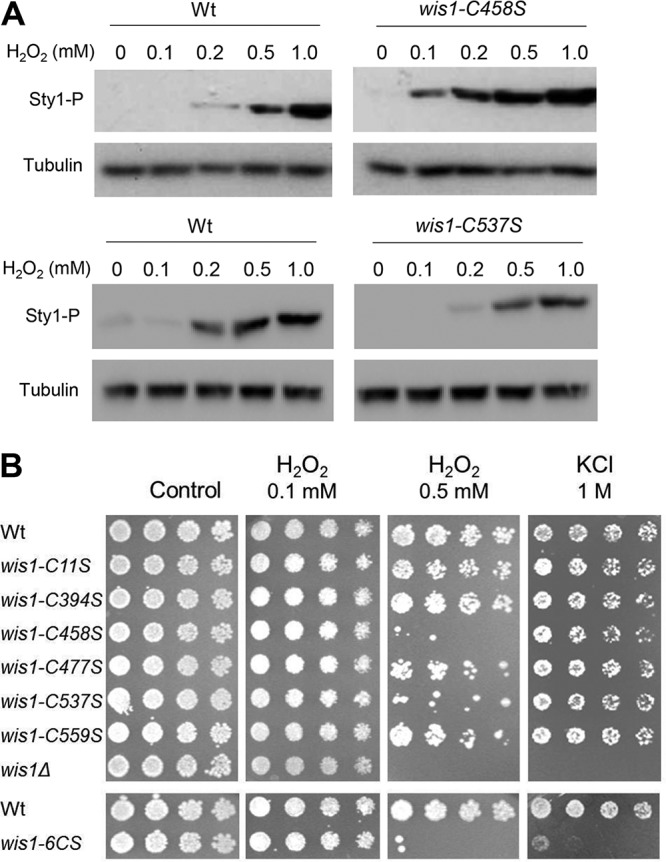
Cys458 inhibits Sty1 phosphorylation (Sty1-P) upon the addition of low levels of H_2_O_2_ and is essential for cellular resistance to H_2_O_2_ but not to hyperosmosis. (A) Lysates from exponentially growing wt (972 *h*^−^), *wis1^C458S^* (JJS18), or *wis1^C537S^* (JJS20) strains, taken before and 20 min after exposure to H_2_O_2_ at the indicated concentrations, were subjected to Western blotting. (B) Exponentially growing cells of wt (972 *h*^−^), *wis1^C11S^* (JJS20), *wis1^C394S^* (JJS17), *wis1C^458S^* (JJS18), *wis1^C477S^* (JJS19), *wis1^C537S^* (JJS20), *wis1^C559S^* (JJS21), *wis1^6CS^* (JJS22), and *wis1Δ* (JJS1) strains were serially diluted and spotted on YES plates with and without 0.5 mM H_2_O_2_ or 1 M KCl.

We next tested the importance of C458 for Wis1 function *in vivo*, first by comparing the H_2_O_2_-induced phosphorylation of Sty1 by Wis1 with that by Wis1^C458S^ (Fig. 4A). Mutants with a C-to-S change at position 458 encoded by *wis1* (*wis1^C458S^*) sustained potent Sty1 phosphorylation, already at a dose that did not trigger wt Wis1 kinase activity, 100 μM H_2_O_2_ (Fig. 4A). Wis1^C458S^-induced Sty1 phosphorylation was also higher than levels of the wt at 200 and 500 μM H_2_O_2_, but at 1 mM the level plateaued at the same intensity as that of the wt. We then tested the effect of the *wis1^C458S^* substitution on the cellular resistance to H_2_O_2_ (Fig. 4B). Interestingly, this mutant was almost as sensitive to H_2_O_2_ as the *wis1*Δ null mutant, whereas it retained wt tolerance to KCl. The *wis1^C537S^* and *wis1^6CS^* mutants were similarly sensitive to H_2_O_2_, whereas substitution of Ser for any of the three remaining Cys residues (C394, C477, and C559) caused only a mild loss of H_2_O_2_ resistance. In contrast to the *wis1^C458S^* substitution, the *wis1^C537S^* mutation decreased the H_2_O_2_-induced phosphorylation of Sty1, suggesting that the impact of this cysteine on Wis1 is distinct from that of C458 and that a balanced Wis1 activation is essential for cells to grow in the presence of H_2_O_2_.

We conclude that C458 is oxidized *in vivo* also in the absence of exogenous applied H_2_O_2_ and thus imparts an inhibitory effect on Wis1 kinase activity, in keeping with the negative effect of the oxidation of C458 by low levels H_2_O_2_ seen *in vitro* (Fig. 2). This increased H_2_O_2_ sensitivity indicates that activation of Sty1 is deleterious at low concentrations of H_2_O_2_.

### The allosteric MEK1 modulator INR119 binds Wis1 close to C458 *in silico*.

**FIG 5:**
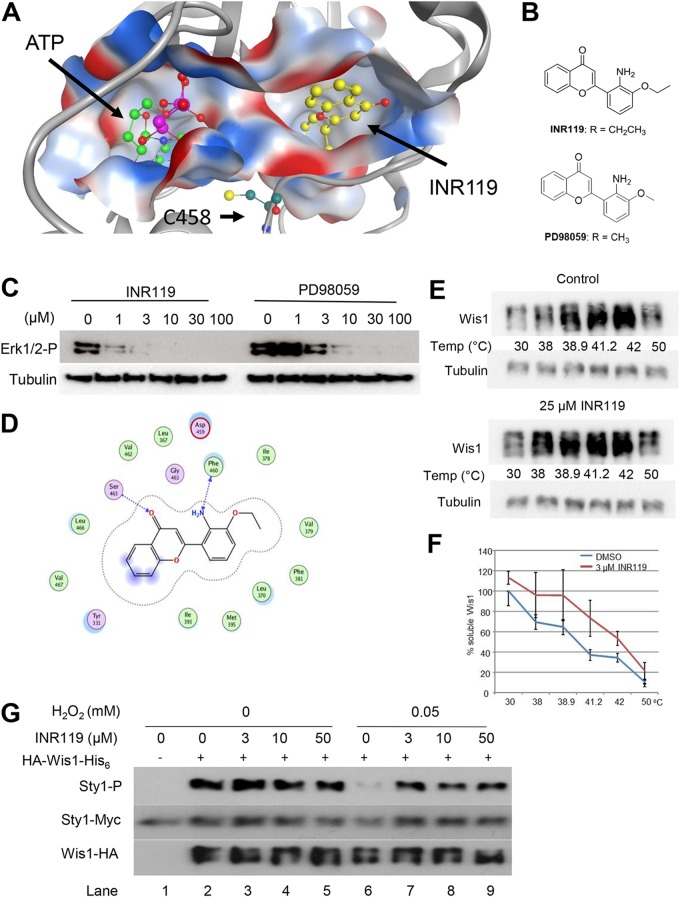
The noncompetitive MAPKK inhibitor INR119 binds Wis1 near C458 and protects Wis1 from inactivation by low levels of H_2_O_2_ *in vitro*. (A) Homology model of Wis1 showing the predicted allosteric pocket with INR119 docked inside and its close proximity to the location of C458 and bound ATP. (B) Structures of INR119 and PD98059. (C) INR119 inhibits the MAPKK MEK1 in human MCF-7 breast cancer cells. Western blots of protein extract from MCF-7 human breast cancer cells exposed for 90 min to different concentrations of INR119 or PD98059 are shown. MEK1 activity was measured as the amount of phosphorylated Erk1/2 (Erk1/2-P). (D) Predictions of interactions between INR119 and the amino acids forming the allosteric pocket. (E) Western blotting of a thermal shift assay performed *in vivo* shows that INR119 stabilizes soluble Wis1 at elevated temperatures, indicating a direct *in vivo* interaction; however, the heat itself also strongly stabilizes soluble Wis1. Live S. pombe cells (JJS6) expressing tagged Wis1 were preincubated with 25 μM INR119 for 15 min and thereafter subjected to the indicated temperature for 3 min. The soluble fraction in lysates was thereafter obtained by centrifugation (see Materials and Methods). (F) Thermal shift assay of the INR119/Wis1 interaction. Cells (JJS6) expressing HA-tagged Wis1 from the *nmt1* promoter were lysed. Cellular extracts were pretreated with 3 μM INR119 for 15 min, and the extracts were heated to the indicated temperatures for 3 min. Soluble protein was separated by centrifugation and analyzed by Western blotting. Quantification of soluble Wis1 is shown at temperatures from 30°C to 50°C. Values have been normalized relative to those of the untreated extract at 30°C and are the averages from four to seven independent experiments. (G) INR119 protects Wis1 from inactivation by low levels of H_2_O_2_ *in vitro*. Semipurified Wis1 (strain JJS7) was pretreated for 15 min with the indicated concentration of INR119 and subsequently for an additional 5 min with 0.05 mM H_2_O_2_. Thereafter the substrate Sty1 was added, as well as MgCl_2_-ATP. Kinase reactions were stopped after 20 min, and results were analyzed by Western blotting.

The MAPKK MEK1 is the closest structural homolog of Wis1 in the mammalian kinome, with 47% amino acid sequence identity. Several allosteric MEK1/MEK2 inhibitors have been reported (16). In general, they bind in a unique pocket adjacent to the Mg-ATP binding site and act by inducing unusual conformations in unphosphorylated MEK1/2, trapping them in a closed but catalytically inactive conformation. A human MEK1 homology-based structural model of Wis1 (PDB code 1S9J) (17) indicates that this pocket is present in Wis1, next to C458 (Fig. 5A). We thus inquired whether allosteric modulators that bind this pocket could modulate the Wis1 H_2_O_2_ response. We tested INR119 among several other allosteric modulators of MEK1 that we had previously produced (12). INR119 was designed as a modification of PD98059 (Fig. 5B), a non-ATP-competitive inhibitor of MEK1 (Fig. 5C) (18). INR119 docked into the allosteric site of Wis1 in the homology-based structural model (Fig. 5A) by forming two hydrogen bonds to the backbone of the protein, between the chromone carbonyl oxygen and the NH group of S483 and between the NH_2_ group of the aniline and the carbonyl oxygen of F460, the phenylalanine in the DFG motif (Fig. 5D). INR119 is thus predicted to bind in the close vicinity of C458 (12), immediately upstream of the DFG motif (19).

To test whether INR119 binds Wis1, we employed a thermal shift assay (20) that measures stabilization of the target protein at elevated temperatures through binding of a ligand. We initially tested binding in live cells, showing that INR119 stabilizes soluble Wis1 at all temperatures tested (Fig. 5E). The elevated temperature itself resulted in massive stabilization of soluble Wis1, precluding calculation of a thermal shift. We therefore tested INR119 binding to Wis1 in crude lysates (Fig. 5F) in which no Wis1 stabilization upon heat was seen, allowing us to calculate a thermal shift of +2°C at 3 μM INR119, in support of a direct interaction.

### INR119 enhances Wis1 activity in response to H_2_O_2_.

**FIG 6:**
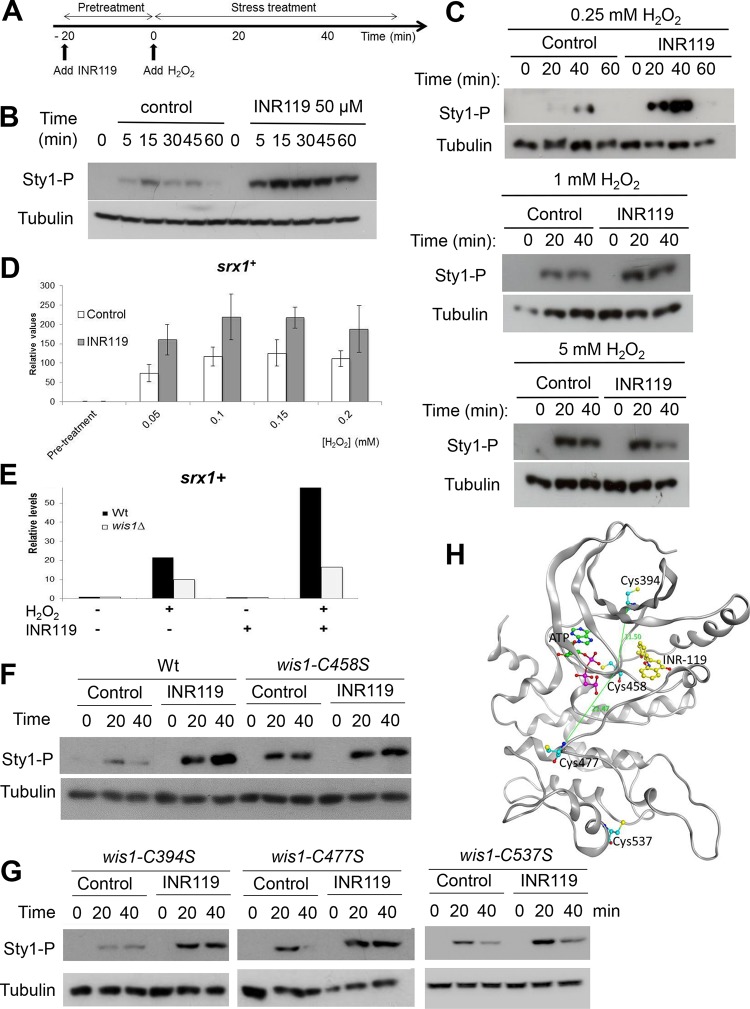
INR119 pretreatment strongly potentiates the Wis1 response to low H_2_O_2_ *in vivo*. (A) Schematic overview of the setup of experiments involving pretreatment with INR119 and H_2_O_2_ addition. (B) Western blot of Sty1 phosphorylation in cells pretreated with 50 μM INR119 and at the indicated time points following the addition 500 μM H_2_O_2_. The time line shows the design of experiment, where INR119 is added 20 min before the onset of peroxide stress. (C) The ability of INR119 to potentiate Wis1 signaling is more pronounced at low levels of H_2_O_2_. Western blotting was performed of cells (972 *h*^−^) pretreated with 50 μM INR119 and following after the addition of different amounts of H_2_O_2_ (0.25 to 5 mM) at the indicated time points as in described for panel A. (D) The Atf1-dependent transcript *srx1^+^* is enhanced by INR119. Wild-type (972 *h*^−^) cells were mock pretreated or pretreated with INR119 for 20 min and thereafter treated with H_2_O_2_ at the indicated concentrations as described for panel A. Samples were taken after 20 min of H_2_O_2_ treatment, and *srx1^+^* transcript levels were determined by qPCR. (E) The *srx1^+^* transcript enhancement by INR119 is Wis1 dependent. Wild-type or *wis1Δ* cells were exposed to 0.2 mM H_2_O_2_ and/or INR119 as indicated, and transcript levels were determined as described for panel D. (F and G) The ability of INR119 to enhance Sty1 activation is dependent on Wis1 C458 (F) but not on C394, C477, or C537 (G). The wild-type (972 *h*^−^), *wis1^C394S^* (JJS17), *wis1^C458S^* (JJS18), *wis1^C477S^* (JJS19), and *wis1^C537S^* (JJS20) strains were analyzed for Sty1 activation by immunoblotting at the indicated time points following the addition of 0.5 mM H_2_O_2_. (H) Structural homology model of Wis1 with the positions of C394, C477, C537, and C458 indicated, as well as the distances between the INR119 binding site and the closest cysteines.

**FIG 7:**
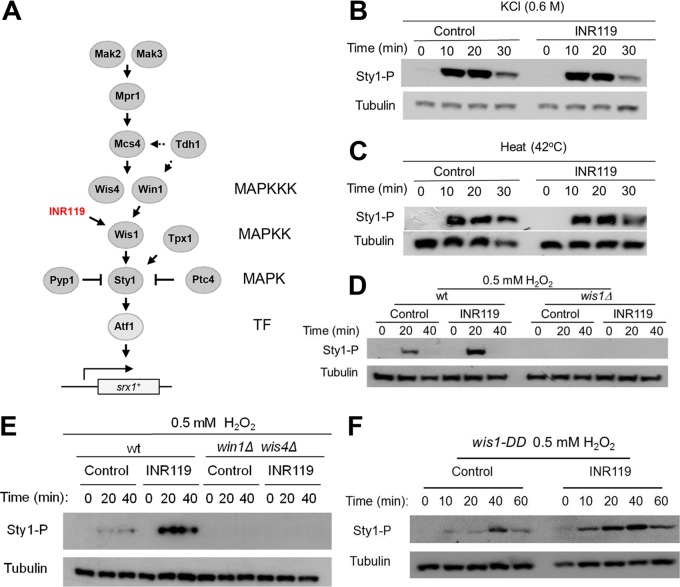
Phosphorylation of Sty1 depends entirely on activated Wis1, and the INR119-induced enhancement of Sty1 pathway activation upon H_2_O_2_ stress requires the upstream activation of Wis1 through the MAPKKKs. (A) Schematic overview of the Sty1 pathway. This simplified schematic summarizes features of pathway architecture relevant for this paper. It is based on information from the literature on protein-protein interactions, modes of pathway activation, and downstream targets (2–8, 23, 25, 45–52). TF, transcription factor. (B and C) INR119 affects neither the response to hyperosmosis (B) nor that to heat (C). Wild-type (972 *h*^−^) cells were mock pretreated (control) or pretreated with 50 μM INR119 for 20 min, and thereafter the culture was treated with KCl (0.6 M) or subjected to heat stress (42°C). (D and E) Sty1 phosphorylation is entirely dependent on Wis1 and on Wis1 activation by the MAPKKKs Win1 and Wis4. Western blots of wt (972 *h*^−^), *wis1Δ* (JJS1), and *win1Δ wis4Δ* (JJS5) deletion mutant cells are shown, as indicated. Cells were pretreated with INR119 and exposed to 0.5 mM H_2_O_2_ as described in the legend to Fig. 4A. The 0-min samples represent cells only pretreated. (F) INR119 induces enhanced Sty1 phosphorylation upon H_2_O_2_ stress also in a *wis1^DD^* strain, with the amino acids targeted by the Win1 and Wis4 MAPKKKs substituted for phosphomimetic amino acids and displaying constitutive Sty1 activation, suggesting that it acts downstream of Wis1 activation by the MAPKKKs. The immunoblot shows Sty1 phosphorylation in a control or INR119-treated *wis1^S469D, T473D^* mutant (22).

We first tested the effect of INR119 on Wis1 kinase activity *in vitro* (Fig. 5G). INR119 did not alter Wis1 activity at concentrations of 3 μM up to 50 μM but, instead, prevented inactivation of Wis1 by H_2_O_2_ (Fig. 5G, lanes 6 to 9; compare, e.g., lanes 2 and 6). We next tested the effect of INR119 on the activity of Wis1 *in vivo* by incubating cells with the inhibitor prior to exposure to H_2_O_2_ (500 μM) (Fig. 6A). Whereas INR119 on its own did not increase Sty1 activity, we observed a strong and Wis1-dependent enhancement of Sty1 phosphorylation upon the addition of H_2_O_2_ (Fig. 6B and 7D). Enhancement was even more potent at low levels of H_2_O_2_ (250 μM) and was then lost at higher levels of the oxidant (5 mM) (Fig. 6C).

To verify whether the effect of INR119 in enhancing the activity of Wis1 translated into expression of the target genes of Atf1, one of the transcription factors regulated by the Wis1-Sty1 pathway, we measured the expression of *srx1^+^* (21) by quantitative PCR (qPCR). INR119 increased *srx1^+^* expression about 2-fold at 50 to 200 μM H_2_O_2_ (Fig. 6D). The effect of INR119 on *srx1^+^* expression was Wis1 dependent as it was not seen in a *wis1*Δ mutant (Fig. 6E). Thus, INR119 potently boosts Sty1 phosphorylation by Wis1 and Atf1-dependent *srx1^+^* expression in response to low doses of H_2_O_2_.

Importantly, the enhancing effect of INR119 was not seen in mutants expressing the Wis1^C458S^ protein, which by itself increased kinase activity (Fig. 6F). This was in contrast to results with Wis1^C394S^, Wis1^C477S^, and Wis1^C537S^, on which the drug exerted the same effect as on the wt protein (Fig. 6G). According to our homology model, C394 and C477, the two cysteines closest to C458, would be too far away from C458 to enable the formation of a disulfide bridge with it (Fig. 6H).

We also investigated the impact of INR119 on Wis1 activity under other stress conditions. Under H_2_O_2_ stress, Wis1 phosphorylation by the MAPKKKs Wis4 and Win1 is crucial for pathway activation (Fig. 7A and E). Interestingly, however, INR119 affected neither the responses to hyperosmotic shock (Fig. 7B) nor those to heat stress (Fig. 7C). The observation that INR119 also did not enhance Wis1 activity in the absence of H_2_O_2_ (Fig. 7B to F) suggested that its enhancing effect is dependent upon the activation of Wis1 by the upstream H_2_O_2_-sensing two-component Mak1/2 system and the MAPKKKs Win1 and Wis4 (Fig. 7A). Indeed, INR119 was not able to stimulate Wis1 activity in the *win1Δ wis4Δ* double mutant (Fig. 7E). However, in contrast, it enhanced the constitutive activity of the *wis1^S469D, T473D^* (*wis1^DD^*) allele, which carries phosphomimetic Asp substitutions of the Ser and Thr residues phosphorylated by Win1/Wis4 (Fig. 7F) (22), in further support of an effect unrelated to but dependent on the activation via the MAPKKKs. INR119 may either prevent the reaction of C458 with H_2_O_2_ or, alternatively, cancel out the inhibitory effect of oxidized C458 on Wis1 activity.

## DISCUSSION

The human p38 pathway is activated by H_2_O_2_ (9), but paradoxically MKK6, the MAPKK upstream of p38, is inactivated by a disulfide bond between C196 (equivalent of Wis1 C458) and C106 and formed in response to low doses of H_2_O_2_ (10). C196 is evolutionarily conserved in mammalian and yeast MAPKKs (Fig. 1A), as well as in several budding and fission yeast MAPKs, but not in other mammalian kinases, which suggests that the redox regulation of C196 is specifically conserved in MAPK pathways (10).

We showed here that the S. pombe MAPKK Wis1 carries the conserved MKK6 Cys residue C196 at position C458 but not MKK6 C106 and is also inactivated by low doses H_2_O_2_ *in vitro* by oxidation of this residue (Fig. 1A and 2D). This conclusion is based on both the reversion of kinase inactivation by thiol reduction, which indicates that inactivation is due to reversible oxidation of a Cys residue, presumably to the sulfenic acid form rather than to a disulfide bond with another Cys residue, and on the effect of the Ser substitution for Wis1 C458, which prevented inactivation. Of note, the reversion of Wis1 inactivation by reduction and its prevention by Ser substitution for Wis1 C458 were both partial, pointing to the presence of other irreversibly modified atomic targets of H_2_O_2_ in Wis1. The mobility shift seen in the mPEG assays in wt Wis1, corresponding to approximately 20 kDa (Fig. 3C, F, and G), further indicates that at least two cysteines are reversibly oxidized.

Even though H_2_O_2_ treatment caused no visible net change of Wis1-oxidized cysteines detectable by the mPEG assay (Fig. 3F and G), there was a rapid increase in sulfenylated Wis1 cysteines (Fig. 3H). This result supports a change in the form of cysteine oxidation in Wis1 upon H_2_O_2_ treatment.

**FIG 8:**
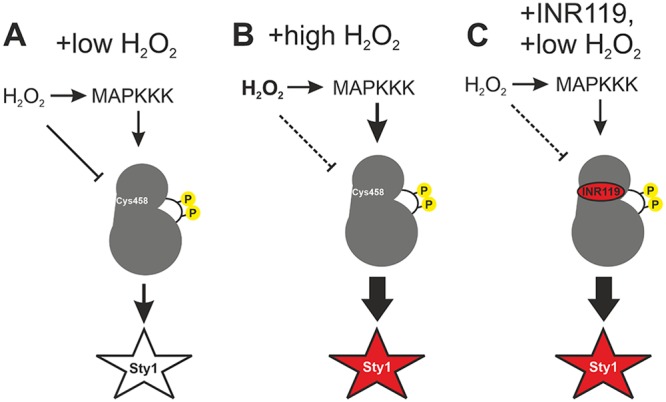
Proposed model of INR119 mechanism of action. (A and B) H_2_O_2_ induces pathway activation through MAPKKK activation; however, at low H_2_O_2_ concentrations negative regulation targeting Wis1 C458 holds the activity of Wis1 back. At higher levels of H_2_O_2_, Sty1 activation is independent of Wis1 C458. (C) INR119 binds Wis1 in an allosteric site next to the active site very close to C458 and protects against negative regulation targeting C458 through stabilizing a conformation unresponsive to negative regulation through C458, resulting in higher Wis1 activity in low H_2_O_2_ levels.

We found that Wis1 oxidized *in vivo* is present also in the absence of exogenous H_2_O_2_ and in part on C458 (Fig. 3C), the substitution of which allowed Wis1 to be activated by H_2_O_2_ at low levels that did not impact the wt enzyme (Fig. 4A). We also found that *wis1^C458S^* mutants are markedly sensitive to H_2_O_2_ but not to KCl (Fig. 4B), pointing to the deleterious effect of unrestrained Wis1 kinase activation, consistent with the toxicity of Wis1 overexpression and Sty1 hyperactivity (6, 23, 24). Altogether, the *in vitro* and *in vivo* data indicate a negative effect of oxidized C458 on Wis1 kinase activity, imposing a threshold that limits undue activation of the pathway at low stress levels (Fig. 8A).

Last, the MEK1 inhibitor INR119, which we show is expected to bind Wis1 close to C458 (Fig. 5A and D), prevented inactivation of Wis1 by H_2_O_2_ *in vitro* and potently boosted the activity of wt Wis1 *in vivo* but not of Wis1^C458S^ (Fig. 6F). Importantly, INR119 did boost the activity of other *wis1* cysteine point mutants, the *wis1^C394S^*, *wis1^C477S^*, and *wis1^C537S^* strains (Fig. 6G). It is noteworthy that, like the *wis1^C458S^* mutant, the *wis1^C537S^* mutant is sensitive to H_2_O_2_ (Fig. 4B), but whereas the *wis1^C458S^* mutant is refractory to kinase activity enhancement by INR119 (Fig. 6F), INR119 still enhances the kinase activity of the *wis1^C537S^* mutant (Fig. 6G). These results further strengthen the notion that INR119 binds in the vicinity of C458 and modulates C458-mediated Wis1 inhibition. Altogether, the *in vitro* and *in vivo* data and the effect of INR119 indicate a negative effect of oxidized C458 on Wis1 kinase activity, imposing a threshold that limits undue activation of the pathway at low stress levels (Fig. 8A). At elevated doses of H_2_O_2_ (≥500 μM), this inhibitory effect may be overcome in the wt enzyme by more potent activation of the upstream pathway (Fig. 8B), which could explain observations of a graded Sty1 pathway response to H_2_O_2_ (25) and identifies Wis1 C458 as an important regulator underlying this property. By changing the conformation of the allosteric site, INR119 may either cancel out the inhibitory effect of oxidized C458 or prevent this oxidation from occurring (Fig. 8C).

The endoplasmic reticulum (ER) transmembrane kinase Ire-1 senses ER stress and activates the unfolded protein response (UPR). In worms and mammals, Ire1 is regulated by sulfenylation of another conserved Cys residue at position +2, relative to the DFG motif (26), which indicates that redox modification of conserved Cys residues located in the kinase activation loop constitutes a conserved mechanism for regulating kinases. Other protein kinases have also been reported to be modulated by thiol oxidation. Notably, oxidation of a conserved cysteine located at DFG +13 in the activation loop (27) of mouse and rat type II protein kinase A catalytic subunits by H_2_O_2_ inhibits kinase activity *in vitro* (28–30). Conversely, protein kinase G and type I protein kinase A (PKA) are both activated *in vitro* through oxidation of the homologous activation loop cysteine upon H_2_O_2_ addition (31, 32). Interestingly, we recently found that a significant proportion of DFG +13 cysteine residues in the catalytic subunit of the S. cerevisiae PKA are glutathionylated *in vivo* in a manner inhibiting PKA activity and increasing cellular H_2_O_2_ resistance (33). Thus, redox modulation of cysteine residues in the activating loop appears to be a conserved means of regulating protein kinases.

Ire1 was shown to be sulfenylated by ER and mitochondrial H_2_O_2_, as well as by H_2_O_2_ produced by a NADPH oxidase in response to arsenite stress, in a manner activating the p38-SKN1 (the worm homolog of NRF2) pathway by, in turn, sulfenylating NSY-1, the worm MAPKKK upstream of p38, brought into the vicinity of Ire1 by the scaffold protein TRAF. Interestingly, Ire1 C663 sulfenylation, or any of the downstream further modified oxidized states of Ire1 C663, totally impaired the ability of Ire1 to induce the UPR (26). The regulation of these two mutually exclusive functions of IRE-1 in the activation of the UPR and of SKN1 by cysteine oxidation of a unique Ire1 Cys residue indicates that, in Wis1, oxidized C458 may similarly afford a qualitatively altered function of this enzyme.

In summary, we showed here that the oxidation of S. pombe MAPKK Wis1 C458 inhibits kinase activity and that this inhibition is required for an appropriate pathway response to peroxide. A possible physiological role of C458 oxidation is to block kinase activation in low levels of endogenous H_2_O_2_. The occurrence of oxidized cysteines in Wis1 under normal growth conditions indicates that endogenously produced H_2_O_2_ would be enough to oxidize Wis1 cysteines including C458. We also showed that the allosteric MEK1 inhibitor INR119 potentiated Wis1 activity by interfering with the inhibitory effect of this residue. In this regard, it is interesting that other allosteric MEK1/2 inhibitors, PD98059, UO126, and PD184352, when used at subinhibitory concentrations prolonged activation of epidermal growth factor (EGF)- and H_2_O_2_-induced MEK5 (34), a kinase also bearing the conserved DFG −1 Cys residue and a side target of allosteric MEK1/2 inhibitors (34, 35), which suggests the presence of a thiol-redox inhibitory mechanism similar to the one of Wis1 also in mammalian cells.

## MATERIALS AND METHODS

### Synthesis of INR119.

INR119 was synthesized as previously described (12).

### Multiple-sequence alignment.

Conservation of MKK6 C109 and C196 was investigated through multiple alignment of human MKK6 against S. pombe, S. cerevisiae, and human MAPKKs, as well as a selection of MAPKs, MAPKKKs, and other serine/threonine kinases in these organisms. Alignment was performed through ClustalO with standard settings, and the result was edited in JalvieW. Cysteine residues (C) are shown in red in Fig. 1 to visualize their conservation.

### Fission yeast strains and growth conditions.

Cells were grown at 30°C in yeast extract with supplements (YES) (36), except for cells overexpressing protein, where instead Edinburgh minimal medium (EMM) was used. S. pombe strains are listed in Table 1.

**TABLE 1:** S. pombe strains and plasmid used in this study

Strain or plasmid	Genotype	Source or reference
Strains		
972	*h*^−^	Lab stock
JJS1	*h*^−^ *wis1*::KanMX6	This study
JJS5	*h*^−^ *win1*::*nat wis4*::KanMX6	This study
JJS6	*h*^−^ KanMX6::*nmt1^+^*::HA_3_::*wis1^+^*	This study
JJS7	*h*^−^ KanMX6::*nmt1^+^*::HA_3_::*wis1^+^* His_6_::NatMX6	This study
JJS9	*h*^−^ KanMX6::*nmt1^+^*::HA_3_::*wis1^C458S^* His_6_::HphMX6	This study
JJS15	*h*^−^ *wis1^+^*-HA_3_::HphMX6	This study
JJS16	*h*^−^ HA_3_::*wis1^C11S, C394S, C458S, C477S, C537S, C559S^* His_6_::HphMX6	This study
JJS20	*h*^−^ *wis1^C11S^* His_6_::HphMX6	This study
JJS17	*h*^−^ *wis1^C394S^* His_6_::HphMX6	This study
JJS18	*h*^−^ *wis1^C458S^* His_6_::HphMX6	This study
JJS19	*h*^−^ *wis1^C477S^* His_6_::HphMX6	This study
JJS20	*h*^−^ *wis1^C537S^* His_6_::HphMX6	This study
JJS21	*h*^−^ *wis1^C559S^* His_6_::HphMX6	This study
JJS22	*h*^−^ *wis1^C11S, C394S, C458S, C477S, C537S, C559S^* ::His_6_::HphMX6	This study
KS1598	*h*^−^ *leu1-32*	53
KS2081	*h*^−^ *wis1^S469, T473D^*::Myc_12_-*ura4^+^*	22
Plasmid pREP41MHNSTY1		46

### Genetic engineering of point mutations.

We designed full or partial *wis1^+^* sequences containing the different substitutions. To engineer missense C-to-S point mutations, the second base in the codon for cysteine was changed from a G to a C, giving TCC or TCT. The designed sequences for construction of all C-to-S-substituted strains all contain a C-terminal His_6_ tag followed by the *hphMX6* hygromycin resistance cassette (37), as well as a 3′ flanking *wis1^+^* sequence. The designed DNA molecules were synthesized and cloned into pMX plasmid vectors by the Thermo Fisher GeneArt service. DNA fragments containing the *wis1* portion were excised from the plasmid vector with the appropriate restriction enzymes and thereafter used to transform S. pombe 972 *h*^−^ to hygromycin resistance and integrated into the endogenous chromosomal *wis1^+^* locus by homologous recombination. Correct integrated sequences and substitutions were confirmed by full gene sequencing.

For construction of JJS16, a strain expressing N-terminally HA-tagged and C-terminally His_6_-tagged Wis1^6CS^ from the endogenous promoter, the following design was used. The sequence started with the sequence upstream of *wis1^+^*, the starting point being a generated SmaI cleavage site, where the original CTTGGG 384 bp upstream of *wis1^+^* had been changed to CCCGGG. As SmaI generates blunt ends, the fragment will not change the original sequence upon integration by homologous recombination. After the start codon, an HA_3_ epitope tag was added, followed by the full *wis1* sequence containing all six C-to-S substitutions, as well as the His_6_ tag before the stop codon. After the hygromycin resistance cassette, a sequence directly downstream of *wis1* followed, ending with a natural NdeI site located 144 bp from the stop codon. The same method was used to obtain a nontagged version of the full *wis1* sequence containing all six C-to-S substitutions, except no HA_3_ epitope tag was added after the start codon. The fragments were cut out from the delivered vector and transformed into 972 *h*^−^, generating JJS16 and JJS22, respectively. Sequences designed to generate strains JJS17 (*wis1^C394S^*), JJS18 (*wis1^C458S^*), JJS19 (*wis1^C477S^*), JJS20 (*wis1^C537S^*), and JJS21 (*wis1^C559S^*), expressing Wis1 C-terminally tagged with His_6_ and with C-to-S substitutions from the endogenous promoter, instead started at a *wis1^+^* internal natural HindIII site. After the *hphMX6* cassette, a sequence downstream of *wis1^+^* ending with a BamHI site was generated by changing GGTAGT 180 bp downstream of *wis1^+^* to GGATCC. Fragments were cut out from the plasmids with HindIII and BamHI and transformed into 972 *h*^−^. For construction of JJS9 (*wis1^C458S^* overexpressed from the *nmt1* promoter), the same fragment used to construct JJS18 was instead transformed into strain JJS6.

### Culture and stress exposure of fission yeast cells.

Unless otherwise stated in the figure legends, cultures growing in mid-log phase were concentrated 20× by mild centrifugation and pretreated with the indicated concentration of INR119 dissolved in dimethyl sulfoxide (DMSO) for the time stated in the figure legend. Controls were pretreated with the corresponding DMSO concentration. At the time of stress induction, pretreated cultures were once again diluted to the original density. Samples representing different time points were harvested through centrifugation at 400 × *g* for 20 s and were thereafter snap-frozen in dry ice.

### Cell lysis and Western blotting.

Unless otherwise stated, cells were lysed by shaking with acid-washed glass beads in a FastPrep FP120 device (Savant) with speed 5 for 30 s. Lysis was performed in lysis buffer A (50 mM NaCl, 50 mM Tris [pH 7.6], 0.2% Triton X-100, 0.25% NP-40, supplemented with phosphatase inhibitor cocktail 04906837001 and protease inhibitor cocktail 04693159001 [Roche]). Protein concentration was determined using a bicinchoninic acid (BCA) assay. Equal protein concentrations of each sample were loaded, and proteins were separated by SDS-PAGE and thereafter blotted onto nitrocellulose membranes.

Phosphorylation of Sty1 was detected by mouse anti-phospho-(Thr180/Tyr182)-p38 antibodies from Cell Signaling Technology (Bionordika AB, Stockholm, Sweden). HA-tagged Wis1 was detected with mouse monoclonal anti-HA (2367; Cell Signaling). The loading control was α-tubulin detected by mouse anti-α-tubulin (T5168; Sigma). Total Sty1 was detected using polyclonal rabbit anti-Hog1 (sc-9079; Santa Cruz Biotechnology) or, for MHNSTY1 (Sty1 with N-terminal Myc and His_6_ tags), with mouse monoclonal anti-c-Myc (9E10, sc-40; Santa Cruz Biotechnology). The secondary antibodies were horseradish peroxidase-coupled anti-mouse A4416 and anti-rabbit A6154 (Sigma).

### Preparation of semipurified protein for *in vitro* kinase assays.

HA-Wis1-His_6_ and MHNSTY1 were expressed separately. HA-Wis1-His_6_ has an N-terminal HA tag and a C-terminal His_6_ tag and is expressed in strain JJS7. The substrate is MHNSTY1 (expressed from plasmid pREP41MHNSTY1 in strain KS1598). As a control, the JJS1 *wis1Δ* strain was used instead of JJS7. Cells were harvested 16 h after induction of the overexpression from the *nmt1* promoters by removing thiamine from the medium. Harvest was done by centrifugation at room temperature (RT) at 850 × *g*, and thereafter cells were resuspended in ice-cold lysis buffer H (50 mM NaCl, 50 mM HEPES, 100 mM KCl, 10% glycerol, 0.2% Triton X-100, 0.25% NP-40, adjusted to pH 7.0, and supplemented with phosphatase inhibitor cocktail 04906837001, protease inhibitor cocktail 04693159001 [Roche], and 10 mM EDTA). All steps from the harvest point until the kinase assay started were carried out at 0 to 8°C. Cells were lysed in a FastPrep FP120 device (Savant) at speed 5 for 40 s. Lysates were cleared of debris through centrifugation for 5 min at 13,000 rpm in a microcentrifuge, and supernatants were further centrifuged for 5 min at 5,000 × *g*. Lysates were thereafter incubated with cOmplete His tag purification resin (28555800; Roche) for 1 h. This resin is compatible with 10 mM EDTA. Beads with HA-Wis1-His_6_ were washed three times with lysis buffer H, and then carefully resuspended beads, now with bound HA-Wis1-His_6_, were aliquoted in equal volumes.

Beads with MHNSTY1 bound were loaded onto a 10-ml plastic column and washed by passing 3 ml of 30 mM imidazole in lysis buffer H through the column. MHNSTY1 was thereafter eluted in lysis buffer H containing 300 mM imidazole by passage four times through the column. Eluted Sty1 was subsequently diluted to lower the imidazole concentration to 30 mM during the kinase assay.

### *In vitro* kinase assays.

Assays were carried out at 30°C and 1,000 rpm in a thermal mixer. Kinase reactions were stopped by addition of sample buffer and denaturing at 95°C for 7 min. The whole volume in each tube was loaded in a single well in a gel, and the result was analyzed by Western blotting.

For kinase assays with H_2_O_2_ and TCEP, resuspended and aliquoted beads with HA-Wis1-His_6_ were treated first for 5 min in pairs with H_2_O_2_, giving the final concentration indicated in the figures, and subsequently for one tube in each pair for 5 min with 1 mM TCEP. Thereafter MHNSTY1 was added, and the kinase reaction was started by addition of MgCl_2_-ATP, giving a final concentration of 20 mM MgCl_2_ (10 mM available for reaction) and 1 mM ATP. Kinase reactions were stopped after 20 min.

For kinase assays with INR119 and H_2_O_2_, resuspended and aliquoted beads with HA-Wis1-His_6_ bound were first pretreated for 15 min with the indicated concentration of INR119 at a final DMSO concentration of 2.5%. As a control, beads treated with 2.5% DMSO alone were used. Subsequently the beads with HA-Wis1-His_6_ bound were incubated for 5 min at the indicated H_2_O_2_ concentration. Thereafter the Wis1 activity against the substrate was investigated by addition of MHNSTY1, as well as by addition of MgCl_2_-ATP, giving a final concentration of 20 mM MgCl_2_ (10 mM available for reaction) and 1 mM ATP. Kinase reactions were stopped after 20 min.

### mPEG assays.

HA-tagged Wis1 protein was expressed from its own promoter (wt strain JJS15 or the *wis1^6CS^* strain JJS16) or was overexpressed from the inducible *nmt1* promoter (strain JJS7 and *wis1^C458S^* strain). Cells were harvested with pure trichloroacetic acid (TCA) added at a final concentration of 20%. Cells were disrupted in a FastPrep FP120 device (Savant) at speed 5 for 40 s, and everything except the beads was then transferred to new tubes. The samples were pelleted by centrifugation and washed three times with ice-cold acetone. Oxidized cysteine residues in the protein extracts were thereafter labeled through the mPEG (63187; Sigma-Aldrich) method, which allows labeling of reversibly oxidized cysteine residues. We used a protocol adapted from Burgoyne et al. (38). Samples were first incubated with 50 mM NEM (04259; Sigma-Aldrich) (in 1% SDS, 100 mM Tris, 10 mM EDTA, pH 7.0) at 37°C for 2 h with agitation (1,400 rpm) to block reduced thiols. Samples were spun down, and the supernatant was precipitated with 20% ice-cold TCA, washed three times with acetone, and dried in a SpeedVac to get rid of residual acetone. The dried samples were next incubated with 50 mM DTT (in 1% SDS, 100 mM Tris, 10 mM EDTA, pH 7.0) at 37°C for 2 h with agitation (1,400 rpm) to reduce the reversibly oxidized thiols. After this incubation step, each sample was divided into two tubes and precipitated and washed with acetone three times. Samples were subsequently incubated either in 2 mM mPEG (in 1% SDS, 100 mM Tris, 10 mM EDTA, pH 7.0) at 37°C for 2 h with agitation (1,400 rpm) for reaction of reduced (initially oxidized) thiols or again blocked by 50 mM NEM. Samples were then precipitated and washed with acetone. For the samples labeled with NEM-NEM-mPEG, the reactions were performed in the following order: NEM-DTT-NEM-DTT-mPEG. Samples (4 to 20 μg protein) were separated by SDS-PAGE. Wis1 protein bands were detected by Western blotting using anti-HA.

### Sulfenic acid labeling.

Cells (50 ml per sample) were grown to an optical density at 600 nm (OD_600_) of 0.7, and H_2_O_2_ was added to the medium to a 0.5 mM final concentration. Samples were taken before and at 30 or 60 s after H_2_O_2_ addition. Cells were collected by centrifugation and resuspended in 0.5 ml of medium per sample. The sulfenyl labeling reagent 4-(pent-4-yn-1-yl)cyclohexane-1,3-dione (DYn-2; Cayman Chemical) was added to 0.5 mM. After 30 min of incubation with gentle mixing at 30°C, cells were collected by centrifugation at 4°C, washed with cold phosphate-buffered saline (PBS), and lysed by vortexing with glass beads in a FastPrep 24. The soluble phase was centrifuged at 14,000 × *g* for 15 min at 4°C, and the supernatant was precipitated twice with 1.2 volumes of a mixture of methanol-CHCl_3_ at 4:1, followed by centrifugation at 14,000 × *g* for 15 min. The interphase containing protein was collected by aspirating away the two liquid phases, washed with cold methanol, left to dry for 10 min, and finally dissolved in 500 μl of PBS–2% sodium dodecyl sulfate (SDS). To the dissolved sample were added biotin azide to 0.5 mM, Cu^2+^-tris((1-benzyl-4-triazolyl)methyl)amine (TBTA) complex to 1 mM, and ascorbate to 2 mM. The samples were incubated in the dark at room temperature for 1 h. After addition of EDTA to 1 mM, incubation was continued for 10 min; samples were precipitated with methanol-CHCl_3_ and centrifuged, and the pellet was washed as described above. Protein was redissolved in 100 mM HEPES (pH 7.4)–2% SDS, and 100 μl of streptavidin-coated beads (Thermo Scientific) was added. After overnight incubation at 4°C, the beads were washed in sequence with 1% SDS 2 times), 4 M urea (2 times), 1 M NaCl (1 time), and water (2 times). Finally, the beads were resuspended in 5× Laemmli buffer, heated to 95°C for 5 min, and separated by SDS-PAGE for immunoblotting.

### Survival assays.

Logarithmically growing cells (972 *h*^−^, JJS1, JJS17, JJS18, JJS19, JJS20, and JJS21) at an OD_600_ of 0.5 were used. Threefold serial dilutions of each strain were spotted on normal YES plates or YES plates containing stress agents (0.5 mM H_2_O_2_, 1 M KCl). The experiment was performed in duplicates. The plates were then incubated at 30°C for 48 h and photographed.

### Homology structural modeling of Wis1 and docking.

The structure of Wis1 was obtained by homology modeling. The protein sequences of Wis1 were obtained from GenBank (accession number NP_595457). The crystal structure of human MEK1 (PDB code 1S9J) (17) was used as the template. The sequence similarity between these two proteins is 47%. The homology model was built using the Structure Prediction Wizard (39) in the Schrödinger Suite (Schrödinger, LLC, New York, NY). The energy-based model-building method was used. The ClustalW method was used to align the target and template sequences in Prime.

For docking, compound INR119 was built in Maestro and prepared by Ligprep in the Schrödinger Suite (Schrödinger, LLC, New York, NY). On the basis of the obtained Wis1 structure from the homology model, the Receptor Grid Generation panel (Schrödinger, LLC, NY) was used to generate grids to specify the position and size of the active site, as well as the allosteric site next to the ATP binding site. To grant full flexibility to the ligands, the XP (extra precision) (40) docking function of Glide (41) was used, and the number of poses per ligand was set to 100. To select the best-docked poses, a postdocking minimization was carried out on the output complexes, and the number of docking poses per ligand for output saving was set at 10.

### *In vivo* MEK1 kinase assay.

MCF-7 breast cancer cells were cultured in Dulbecco’s modified Eagle medium (DMEM) supplemented with 10% fetal calf serum, 0.5% l-glutamine, and 0.5% PenStrep (Gibco/Life Technologies). Cells were grown at 37°C in a humidified atmosphere containing 5% CO_2_.

Nonstarved cells were treated for 90 min with either DMSO alone or different concentrations (2 to 100 μM) of INR119 dissolved in DMSO. Cells were lysed by addition of HEPES lysis buffer (50 mM HEPES [pH 7.5], 10 mM NaCl, 1% Triton X-100, 10% glycerol, 5 mM MgCl_2_, 1 mM EDTA) supplemented with phosphatase inhibitor cocktails P5726 and P0044 (Sigma-Aldrich) and protease inhibitor cocktail 04693159001 (Roche), together with incubation at 4°C with gentle shaking for 30 min. Phosphorylation of Erk1/2 was analyzed by Western blotting as described previously (42).

### Thermal shift assays.

S. pombe strain JJS6 expressing Wis1 with an N-terminal HA_3_ tag from the inducible *nmt1^+^* promoter integrated in the chromosomal *wis1^+^* locus was grown for 24 h in EMM without thiamine to a final OD_600_ of 2.0 to induce maximal expression of Wis1. For each sample, 6.4 × 10^8^ cells were used. For thermal shift assays in live cells, cells were subjected to 15 min of 25 μM INR119 pretreatment and then heated to the temperatures indicated in Fig. 5E for 3 min. Subsequently, the temperature was lowered to 25°C for 3 min before snap-freezing cells on dry ice. Cells were thereafter thawed on ice and lysed by shaking with acid-washed glass beads in a FastPrep FP120 device (Savant) at speed setting 5 for 30 s. Lysis was performed in ice-cold lysis buffer A (50 mM NaCl, 50 mM Tris [pH 7.6], 0.2% Triton X-100, 0.25% NP-40) containing phosphatase inhibitor cocktail 04906837001 and protease inhibitor cocktail 04693159001 (both from Roche). Lysates were subjected to centrifugation at 20,000 × *g* for 20 min to separate soluble and insoluble proteins. Finally, the supernatants containing soluble protein were analyzed by Western blotting.

For thermal shift assays on crude extracts, cells were instead first lysed as described above and then subjected to a 5-min centrifugation at 13,000 rpm in a microcentrifuge to get rid of foam. Protein lysates were thereafter preincubated with 3 μM INR119 for 15 min and then heated to the temperatures indicated in Fig. 5F for 3 min. Subsequently, the temperature was lowered to 25°C for 3 min before snap-freezing lysates on dry ice. Frozen lysates were thawed on ice and subjected to centrifugation at 20,000 × *g* for 20 min to separate soluble and insoluble proteins. Finally, the supernatants containing soluble protein were analyzed by Western blotting.

### Quantitative RT-PCR.

Lysates were prepared from exponentially growing cultures that were incubated under the conditions and times indicated in Fig. 6D and E and in the figure legend. RNA was isolated, and quantitative reverse transcription-PCR (RT-PCR) was performed as described previously (43) using primers qsrx1F (5′-GCTCACGATGAAGCAGGGCG-3′) and qsrx1R (5′-GGCGTAGAGTGTTAGGGGAGCA-3′). Primer pairs for reference genes *act1^+^* and *nda3^+^* were the following (in respective order): the pair qact1F (5′-CCGTGCCCCTGAAGCTCTTT-3′) and qact1R (5′-GCCTCATGAATACCGGCGTTT-3′) and the pair qnda3F (5′-AATATGATGGTCGCCGCTGA-3′) and qnda3R (5′-ACGGAAAAGAGCGGCAACTG-3′). Data and errors bars represent the averages and standard deviations of four independent biological samples.
